# Evaluating YouTube as a Good Resource for Enhancing Patient Understanding of Heart Failure

**DOI:** 10.7759/cureus.68243

**Published:** 2024-08-30

**Authors:** Emre Yılmaz, Sencer Çamcı, Fatih Özçubukçu, Aziz Şahin

**Affiliations:** 1 Cardiology, Giresun University Faculty of Medicine, Giresun, TUR

**Keywords:** online videos, youtube, social network, quality, heart failure

## Abstract

Objective: The aim of our study was to evaluate the content and quality of heart failure posts on the video-sharing site YouTube, which is an easily accessible source of information and is becoming increasingly popular in society for obtaining health information.

Methods: In December 2023, we evaluated 162 videos after applying the exclusion criteria as a result of our search with the keyword "Heart Failure" on English-sharing sites. In addition to the technical data of the videos, such as views, duration, upload day, likes, and dislikes, we also used indices such as power index and popularity score in the analyses. We evaluated the quality of the videos using the DISCERN and global quality score scales, and the content using our content score scale. We classified the videos in the study into three quality subgroups according to the scores they received on all three scales.

Results: The median number of views of the videos included in our study was 31092 (interquartile range (IQR): 3929-127758) and the median video duration was 336 (IQR: 189-843) seconds. The median popularity score was 28.25 (IQR: 4.7-143) and the median power index was 35139 (2061-308128). Group 1 (low quality) included 54 videos with a total score between 10 and 14, group 2 (medium quality) included 54 videos with a total score between 15 and 22, and group 3 (high quality) included 54 videos with a total score between 23 and 30. Views, upload day, and video duration were significantly higher for group 3 videos (p = 0.008, p = 0.001, and p < 0.001, respectively). Likes and dislikes were not significantly different between groups. The popularity score was significantly higher for group 1 videos (39.5 (IQR: 6.5-200), p = 0.023), while the power index was significantly higher for group 3 videos (74206 (IQR: 9477-221408), p = 0.006).

Conclusions: Our findings confirm that YouTube, a video-sharing website, is essential for easily sharing and spreading health-related information to a broad audience. Increased attention to videos with scientific content and high-quality scores suggests that YouTube provides accurate and quality information about heart failure. While the number of quality posts tends to increase daily, healthcare professionals should be encouraged to share high-quality scientific videos more frequently.

## Introduction

A significant increase in the use of the internet, including platforms such as YouTube (YouTube, LLC, San Bruno, USA), for accessing health-related information has been revealed by studies. Approximately eight out of 10 internet users utilize online resources for this purpose [1,2]. YouTube, a rapidly growing online video platform, garners 100 million viewers and nearly two billion views daily [3,4]. A national health survey conducted in 2018 revealed that over 33% of patients utilize YouTube to access health-related videos [5]. However, the platform users, viewers, and uploaders are not homogeneous. Furthermore, the quality or content of online videos is not subject to approval. This situation can facilitate the spreading of false or misleading information, including health information [4,5].

Heart failure is a chronic, progressive condition characterized by weakening symptoms, reduced quality of life, and limited survival [6,7]. It is a significant burden on patients and healthcare systems. Heart failure is a worldwide epidemic affecting more than 26 million people [8]. Furthermore, it is estimated that heart failure has an economic cost to healthcare systems of $108 billion annually [9]. Furthermore, the burden of heart failure on healthcare systems is compounded by the occurrence of recurrent hospitalizations, prolonged hospitalizations, and intensive care needs during decompensation periods [10].

Over the past few years, YouTube videos have become a popular way for individuals and health professionals to obtain information, and researchers have evaluated the qualities and reliability of these videos. However, no study to our knowledge has assessed the quality of YouTube videos on heart failure. The aim of this study was to investigate the scientific evidence, credibility, and quality of English-language heart failure videos uploaded to YouTube.

## Materials and methods

The present study was conducted on YouTube (http://www.youtube.com) to identify the most popular heart failure videos. First, the "ChatGPT-3.5" (https://chat.openai.com) artificial intelligence-based solution tool was used to determine the keyword suggestions received by YouTube users about heart failure. The keyword suggestions are presented in Table 1.

**Table 1:** Keyword suggestions received by ChatGPT-3.5 for YouTube searches on "heart failure" and "heart failure in adults"

Keyword suggestions received by ChatGPT-3.5
"Heart failure"
"Congestive heart failure"
"Understanding heart failure"
"Symptoms of heart failure"
"Causes of heart failure"
"Treatment for heart failure" = "Heart failure treatment"
"Living with heart failure"
"Heart failure management"
"Heart failure diagnosis"
"Preventing heart failure"
"Cardiac rehabilitation for heart failure"
"Heart failure diet and nutrition"
"Heart failure medications"
"Heart transplant for heart failure"
"Heart failure prognosis"
"Heart failure support groups"
"Latest research on heart failure"
"Patient stories of heart failure"
"Heart failure in elderly"
"Heart failure in adults"

The frequency of use of these keywords was evaluated using Google Trends (http://www.google.com/trends/; Google, LLC, Mountain View, USA) [11]. The keyword "Heart Failure" was identified as the most prominent in the YouTube scans conducted between January 2008 and December 2023 globally. While 100 points can be obtained in the interest scoring over time, "Heart Failure" was the most popular keyword, averaging 54 points. It was followed by "Congestive Heart Failure," with an average of 11 points. Other keywords were ≤1 point. Considering the considerations above, a YouTube search was conducted in December 2023 using the keyword "Heart Failure." The first 10 pages of search results (n: 10 * 20 = 200) were evaluated, each containing 20 videos. This evaluation was carried out by similar studies in the literature [11,12]. At this juncture, a two-step evaluation process was initiated. In the initial step, the videos were subjected to a preliminary assessment, which evaluated the title, promotional text, and the first 30 seconds of viewing. In the subsequent step, all videos underwent a comprehensive examination, during which their quality and content were assessed.

After a preliminary evaluation, the first 200 videos were analyzed according to inclusion and exclusion criteria based on previous examples in the literature to obtain the sample for this study [13,14].

Videos about heart failure that were longer than 30 seconds and had English audio were included. Videos with good image and sound quality, clear and comprehensible language, appropriate information for calculating content scores (CS), and comprehensible images were preferred for inclusion. Videos with poor or no sound and videos on other topics were excluded. If all or part of the same video was posted by different users, the main video was included for assessment, and parts or copies of the same video were excluded from the study. Eleven videos with low-quality audio or no audio, 14 videos on other topics, six videos <30 seconds, and seven videos not in English were excluded from the study. As a result, a total of 162 videos published between December 2008 and December 2023 were included in our analyses.

To minimize biases in the YouTube search algorithm for videos based on the localization of the study computer and search activity, we performed all searches on a single day using the incognito mode of the Google Chrome browser (Google, LLC). We saved the results for later analysis [15]. The search results were assessed by two independent cardiologists (E.Y. and F.Ö.). Kappa analysis was applied to evaluate "intra-observer agreement" by watching and scoring the same videos again by the same observer one week apart and "inter-observer agreement" by evaluating and scoring the same video by two observers.

From the included videos, the following descriptor data were collected: the video duration (in seconds), the time from the upload date of the video to the search date ("upload days"), the popularity score, and the number of views, likes, and dislikes. Popularity score was calculated by the formula: number of views on the search date/"upload days" [4,16,17]. As video sources, medical dot-com channels (MDC) (i.e., institutional channels with an external internet address shared by multiple healthcare professional users and where additional information can be accessed), independent user channels (IUC), news agency channels (NAC), and hospital-university channels (HUC) were used. The video power index was calculated with the formula: (number of views*number of likes)/100 [18].

We developed a CS for the content analysis of the videos. For this purpose, we conducted a review using a binary coding system (0 and 1 points) for the following 20 topics or categories: epidemiology, pathogenesis, symptoms, diagnosis, exercise, diet, drug therapy, family/social life, and support, screening, prevention, acute heart failure, dyslipidemia, hypertension, smoking cessation, complications (including coronary heart disease, renal disease, arrhythmias), immunization, hospitalization, implantable device therapies, weight management and mental health. If the video content presented the correct information on the relevant topics, a "1" point was given in this category and expressed as a quality score. If the topic was not mentioned or if incorrect information was presented, the video was scored "0" in this category. It was planned that videos would receive a minimum score of "0" and a maximum score of "20" as a result of CS. The accuracy of the topics and video content in the coding scheme was determined by reference to the European Society of Cardiology (ESC) 2021 guidelines for the diagnosis and management of acute and chronic heart failure [19].

A scoring system adapted from the DISCERN tool called the DISCERN score (DS), was used to evaluate the reliability of the information provided in the videos selected. This scale consists of five questions with a "yes" or "no" answer. Each "yes" answer was calculated as 1 point (indicates high confidence) and each "no" answer was calculated as 0 points (indicates low confidence) [16,17,20,21] (Table 2).

**Table 2:** DISCERN score

DISCERN score
1. Are the educational goals clearly stated and achieved?
2. Are reliable sources of information used?
3. Is the information presented balanced and unbiased?
4. Are additional sources of information listed for users to refer to?
5. Are areas of uncertainty, gaps, or differences of opinion mentioned?

In addition, the videos were evaluated for overall ease of interpretation and flow of information using the global quality score (GQS), which was used to rate the overall quality of each video (scored from 1-5, where 1 indicates low quality and 5 indicates excellent quality) [22,23] (Table 3).

**Table 3:** Global quality score

Global quality score
1. Not useful at all for viewers.
2. Poor quality in general and poor video streaming; very limited use is recommended for viewers.
3. Medium quality and insufficient flow; some important information is provided, but other information is missing. A little useful for the audience.
4. Good quality and generally good flow; most of the relevant information is listed, but some topics have not been covered; useful for viewers.
5. Excellent quality and perfect flow; very useful for viewers.

We divided the study videos into three equal groups according to the distribution of CS + DS + GQS score totals. Group 1 (low quality) included 54 videos with total scores between 10 and 14, group 2 (medium quality) included 54 videos with scores between 15 and 22, and group 3 (high quality) included 54 videos with scores between 23 and 30.

Ethical approval

Public YouTube videos were analyzed and no human participants or animals were included in the study, so ethical approval was not required, similar to other YouTube studies [12,24].

Statistical analysis

The IBM SPSS Statistics for Windows, Version 22 (Released 2013; IBM Corp., Armonk, New York, USA) was used for all statistical analyses. To evaluate the study data, descriptive statistics (mean, standard deviation, median, frequencies, and percentages) were used. The Kolmogorov-Smirnov test was used to determine the normality of quantitative data. For categorical variables, the chi-squared test was used. Continuous variables of the quality groups were compared by one-way ANOVA analysis. Significant differences between groups were assessed by post hoc Tukey and Games-Howell analyses. To measure intraobserver (one week apart) and interobserver agreement, the kappa statistic was used. For all analyses, a p-value less than 0.05 was considered statistically significant.

## Results

**Figure 1:**
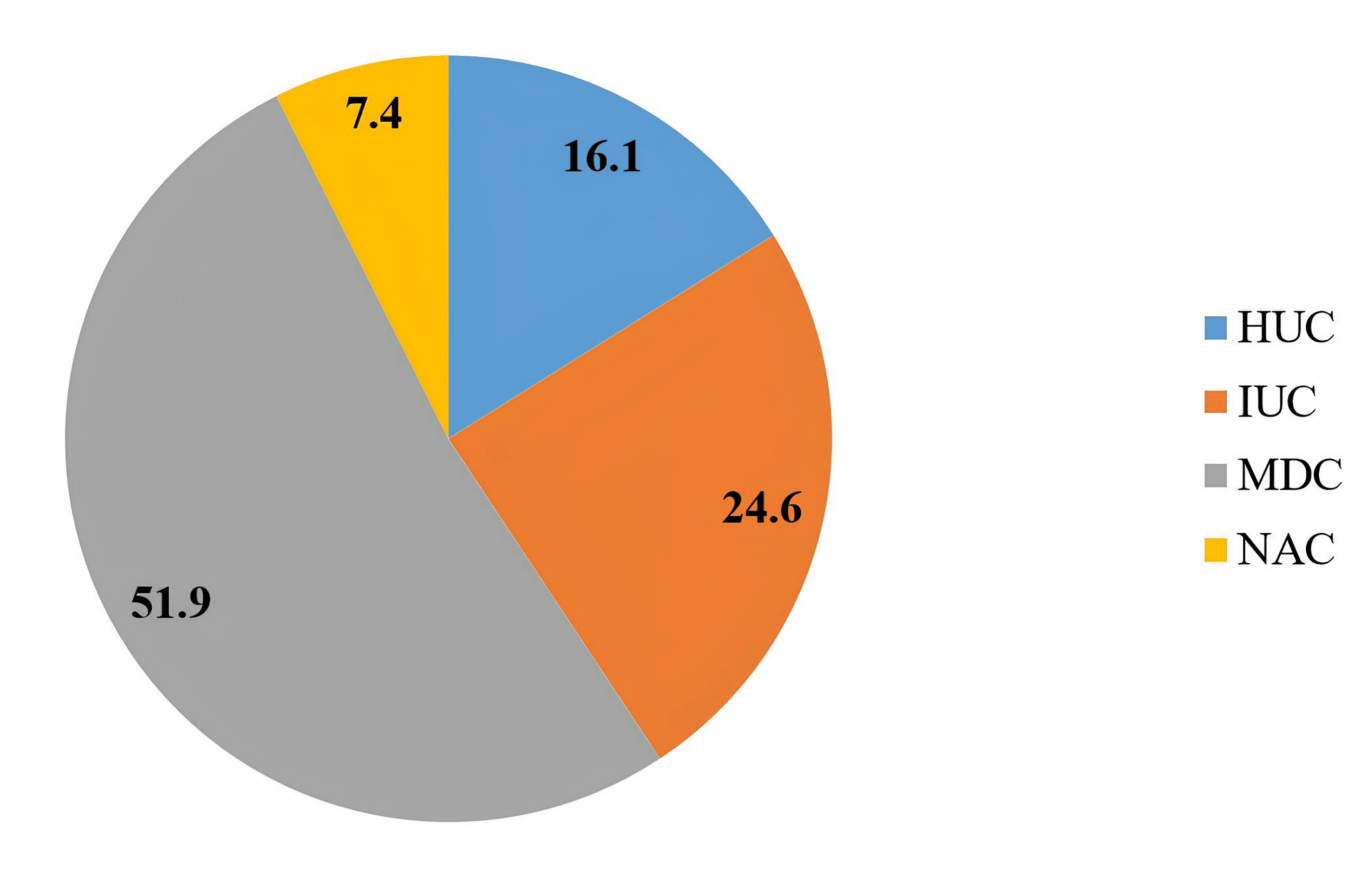
Distribution of video resources MDC: medical dot-com channels; IUC: independent user channels; NAC: news agency channels; HUC: hospital-university channels

Of the 162 videos evaluated in our study, the median number of views was 31092 (interquartile range (IQR): 3929-127758), the median upload day was 1002 (IQR: 543-2962) days, and the median video duration was 336 (IQR: 189-843) seconds. For video interactions, the median number of likes was 97 (IQR: 27-476) and the median number of dislikes was 5 (IQR: 0-34). The median popularity score was 28.25 (IQR: 4.7-143) and the median power index was 35139 (2061-308128). About 26 (16.1%) videos originated from HUC, 40 (24.6%) from IUC, 84 (51.9%) from MDC, and 12 (7.4%) from NAC (Figure 1). The results of the quality and content analyses showed a median DS of 3 (IQR: 2-4), a median GQS of 3 (IQR: 2-4), and a median CS of 9 (IQR: 5-16) (Table 4).

**Table 4:** Demographic data of study videos IQR: interquartile range; HUC: hospital-university channel; IUC: independent user channel; MDC: medical dot-com; NAC: news agency channel; popularity score: view/upload day; power index: (view*likes)/100

Variables	All (n = 162) Median (IQR)
Views	31092 (3929-127758)
Upload day, day	1002 (543-2962)
Likes	97 (27-476)
Dislikes	5 (0-34)
Popularity score	28.25 (4.7-143)
Duration, seconds	336 (189-843)
Power index	35139 (2061-308128)
HUC, n (%)	26 (16.1%)
IUC, n (%)	40 (24.6%)
MDC, n (%)	84 (51.9%)
NAC, n (%)	12 (7.4%)
DISCERN score	3 (2-4)
Global quality score	3 (2-4)
Content score	9 (5-16)

In our study, we made video quality subgroups according to DS + GQS + CS score totals. The three groups were divided equally according to the total score distributions and named group 1 (low quality), group 2 (moderate quality), and group 3 (high quality). View, upload day, and video duration were significantly higher in group 3 videos (p = 0.008, p = 0.001, and p < 0.001, respectively). Likes and dislikes did not differ significantly between the groups. Popularity score was significantly higher in group 1 videos (39.5 (IQR: 6.5-200), p = 0.023), while power index was significantly higher in group 3 videos (74206 (IQR: 9477-221408), p = 0.006). HUC and NAC-derived videos were significantly higher in group 3, while IUC-derived videos were higher in group 1. The distribution of MDC-derived videos between the groups did not cause a significant difference. Subgroup analysis details are presented in Table 5.

**Table 5:** Comparison of video quality subgroups IQR: interquartile range; HUC: hospital-university channel; IUC: independent user channel; MDC: medical dot-com; NAC: news agency channel; popularity score: view/upload day; power index: (view*likes)/100 ^^^ denotes statistical significance with a p-value less than 0.05

Variables	Group 1 (low quality) (n = 54) Median (IQR)	Group 2 (medium quality) (n = 54) Median (IQR)	Group 3 (high quality) (n = 54) Median (IQR)	p-value
Views	37310 (4717-133309)	27982 (3143-102206)	49747 (6679-207188)	0.008^^^
Upload day, day	901 (434-2349)	1302 (641-3524)	1823 (977-5230)	0.001^^^
Likes	81 (22-404)	103 (25-422)	97 (24-398)	0.284
Dislikes	5 (0-34)	5 (0-32)	7 (4-32)	0.109
Popularity score	39.5 (6.5-200)	31 (5-132)	24 (4.1-128)	0.023^^^
Duration, seconds	140 (107-364)	281 (133-724)	443 (176-1104)	<0.001^^^
Power index	48523 (16874-148578)	32047 (10549-98242)	74206 (9477-221408)	0.006^^^
HUC, n (%)	0	7 (13%)	19 (35.2%)	<0.001^^^
IUC, n (%)	24 (44.4%)	10 (18.5%)	6 (11.1%)	
MDC, n (%)	30 (55.6%)	33 (61.1%)	21 (38.9%)	
NAC, n (%)	0	4 (7.4%)	8 (14.8%)	

Interobserver and intraobserver agreement for the three scoring systems used in our study were evaluated by kappa analysis. Interobserver agreement (for E.Y. and F.Ö.): kappa statistic value for DS was 0.81 (p = 0.011), kappa statistic value for GQS was 0.79 (p = 0.009), and kappa statistic value for CS was 0.94 (p < 0.001). Intraobserver agreement (for E.Y.): kappa statistic value for DS 0.83 (p = 0.023), kappa statistic value for GQS 0.78 (p = 0.005), and kappa statistic value for CS 0.90 (p < 0.001). Intraobserver agreement (for F.Ö.): kappa statistic value for DS 0.80 (p = 0.007), kappa statistic value for GQS 0.80 (p = 0.001), and kappa statistic value for CS 0.90 (p < 0.001).

## Discussion

YouTube is becoming increasingly popular with patients due to its quick visualization and easy access to information [25,26]. Data are showing that obtaining information from the internet supports patients' participation in health decisions, healthy lifestyle changes, and medication adherence. However, patients' shortcomings in assessing the accuracy of this easily accessible information may lead to various problems in the patient-doctor relationship, quality of care, and health outcomes. The aim of our study was to evaluate the quality and content of YouTube videos on heart failure in terms of patient education [27].

The key findings of our study are as follows: 66.6% of YouTube videos on heart failure presented moderate to high-quality content. Some authors preferred to categorize video content and quality as useful or misleading. For example, Kaya et al. [28] reported that 50.9% of the videos they evaluated were useful in their study of hypertension and its treatment. On the other hand, Kumar et al. [20] reported that 63% of the videos they evaluated in their YouTube analysis on hypertension provided useful information. This numerical difference between authors may be due to methodological differences. The method of sampling and the definition of usefulness are important in this difference. Kaya et al. searched six different evaluation titles and included the videos on the first three pages of each search result in the preliminary evaluation of their study. This method resulted in many duplicate videos and reduced the sample size. Kumar et al. used the same sampling method that we used in our study. Restricting the search term and pre-evaluating the first 10 pages may reduce the likelihood of encountering duplicates and provide the opportunity to access a greater variety of videos. We used a different methodology than the authors regarding the usefulness of the videos. The authors defined usefulness as consistency with scientific data and preferred to analyze the videos in two groups as useful and misleading. However, concerned that this grouping was not suitable for detailed analysis, we preferred to evaluate the videos in three equal groups according to the total score they received from the quality and CS.

Of the videos we rated, 51.9% were from MDC. About 64.2% of MDC-originated videos were rated in the medium and high-quality groups. HUC-originated shares accounted for 16.1% of the videos we rated, and NAC-originated shares accounted for 7.4%. We found that the videos of both sharing groups were in the medium and high-quality groups. IUC-originated shares represented 24.6% of the videos we evaluated. We rated 60% of their posts in the low-quality category. In a YouTube analysis on heart failure, Eliya et al. [29] reported that of the videos they analyzed, 64.2% were shared by institutions, 28.5% by healthcare professionals, and 7.3% by patients. Although our definitions of video sources are different, it can be seen that the sharing rate of non-health professional groups, which we characterize as IUC or patients, is low. The authors preferred to assess the content rather than the quality of the patient-generated videos they identified. They reported that the majority of videos shared by this group focused on patient empowerment, support, and awareness. In our study, we found that most ICU videos provided misleading and incomplete information. The most common criterion used to evaluate the quality of YouTube videos on health-related information and education is the rating and review by health professionals and experts [30]. In the scientific publishing culture, it is essential for us researchers to refer to accepted information that has been proven to be accurate and valid in the literature and to find the most accurate information through peer review. Similarly, the evaluation of YouTube content by health professionals and experts increases the accuracy and reliability of the information presented. However, this is not a sustainable option on a platform such as YouTube, where the volume of content is growing exponentially. Therefore, YouTube content produced by health professionals and experts seems to be one of the most appropriate ways to access accurate and reliable health information. Our findings support this conclusion.

If we talk about our technical data; the number of views was significantly higher in group 3. In our quality sub-grouping, we evaluated video streaming and visuality levels along with content. We think that the number of views may be higher because these characteristics of the group 3 videos are superior to the other groups. This result is consistent with similar studies in the literature [20,31]. The high number of views of videos with good scientific quality is an indication of the viewer's interest in and preference for accurate information. On the other hand, the duration of the upload day for group 3 videos was significantly higher than for the other groups. The popularity of videos is inversely proportional to the upload day and directly proportional to the number of views. In other words, the more attention a video receives in a short period after it is published, the more popular it is. In our analyses, we found that the popularity score was significantly higher for group 1 videos. Unfortunately, this result supports the fact that the speed at which high-quality videos reach a large audience is still not sufficient. The popularity score is an index that aims to evaluate how much attention videos receive in a short period. The fact that this score is significantly higher for group 1 videos raises concerns about the rate at which misleading or incomplete information is disseminated. One of the possible reasons for this could be that the video duration is shorter in this group of videos. Gabarron et al. [30] reported that popularity was the second most frequently cited concept for assessing quality on YouTube. In their review, popularity was often expressed in terms of number of views and/or overall rating. For this reason, we used the popularity score, which corresponds to the average number of daily views, and the power index, which evaluates the number of views together with similar data from the general rating criteria. Although these data are often preferred for quality evaluation, they should be interpreted with caution. This is because these data can be manipulated due to the viral effect of YouTube, which can occur both with marketing strategies and by sharing the video in question on different websites.

We found no significant difference between our quality groups in terms of the distribution of likes and dislikes. These parameters are an indicator of the interaction of the videos with the viewers. On the other hand, another index we use to evaluate this interaction is the power index. This index, which is obtained by multiplying the number of views by the number of likes, was found to be higher for group 3 videos. We can interpret this result in the direction that quality posts get the interaction they deserve. In a previous study analyzing YouTube videos related to echocardiography, we reported a significant positive correlation between the number of views and likes and between the power index and the quality scales [12]. This result also supports the information in the literature.

In the quality groups, we found that video duration increased significantly as the quality level increased. The aim of our study was to assess the quality and content of the videos we evaluated in terms of patient education about heart failure. This result highlights the need for the content of the video to reach a certain level of quality.

In conclusion, the quality and content of heart failure videos on YouTube are above average. We recommend that the HUC, NAC, and MDC groups, which produce high-quality content, should be encouraged to increase the number of posts to reach a wider audience with higher quality and more accurate information. These groups should also be supported by social media experts to produce content in the most appropriate format for the audience in light of the literature on the subject, which can increase the popularity and power of accurate information.

Limitations

The focus on English-language videos and the use of a cross-sectional study design are the main limitations. According to YouTube's press statistics, 48 hours of video are uploaded to the system every minute. This means that about eight years' worth of content is uploaded every day. Thus, the information published on the site fluctuates significantly over time (YouTube.com. Pressroom statistics. http://www.youtube.com/ t/press_statistics; accessed January 19, 2012). YouTube also continues to grow in popularity and accessibility, which means that conducting scientific research on such a platform needs to be updated frequently. Furthermore, video optimization and analysis can lead to some variability in search results for the same keyword between two users, due to the dynamic content and variable algorithm used by YouTube. We tried to mitigate this limitation by analyzing a large number of videos. Viewers' interactions with the videos were evaluated directly on the YouTube website. We did not evaluate YouTube videos that were shared on other websites or social media platforms. Due to changes in guideline recommendations, the large number of videos evaluated in our study (2008-2023) may limit our results.

## Conclusions

Our findings confirm that YouTube, a video-sharing website, is essential for easily sharing and spreading health-related information to a broad audience. Increased attention to videos with scientific content and high-quality scores suggests that YouTube provides accurate and quality information about heart failure. While the number of quality posts tends to increase daily, healthcare professionals should be encouraged to share high-quality scientific videos more frequently.
